# Prospective Evaluation of Complications and Associated Risk Factors in Breast Cancer Surgery

**DOI:** 10.1155/2022/6601066

**Published:** 2022-09-17

**Authors:** Linda Adwall, Hella Hultin, Maria Mani, Olov Norlén

**Affiliations:** ^1^Department of Surgical Sciences, Uppsala University, Uppsala, Sweden; ^2^Department of Molecular Medicine and Surgery, Karolinska Institute, Stockholm, Sweden

## Abstract

**Background:**

Surgical site infection (SSI) is a well-known complication after breast cancer surgery. The primary aim was to assess risk factors for SSI. Risk factors for other wound complications were also studied.

**Materials and Methods:**

In this prospectively registered cohort study, patients who underwent breast-conserving surgery (BCS) or mastectomy between May 2017 and May 2019 were included. Data included patient and treatment characteristics, infection, and wound complication rates. Risk factors for SSI and wound complications were analyzed with simple and multiple logistic regression.

**Results:**

The study cohort consisted of 592 patients who underwent 707 procedures. There were 66 (9.3%) SSI and 95 (13.4%) wound complications. “BMI > 25,” “oncoplastic BCS,” “reoperation within 24 hour,” and “prolonged operative time” were risk factors for SSI with simple analysis. BMI 25-30 and >30 remained as significant risk factors for SSI with adjusted analysis. Risk factors for “any wound complication” with adjusted analysis were “mastectomy with/without reconstruction” in addition to “BMI 25-30” and “BMI > 30.”

**Conclusion:**

The only significant risk factor for SSI on multivariable analysis were BMI 25-30 and BMI > 30. Significant risk factors for “any wound complication” on multivariable analysis were “mastectomy with/without reconstruction” as well as “BMI 25-30” and “BMI > 30”.

## 1. Introduction

In 2020, there were 2.3 million women diagnosed with breast cancer and 685 000 deaths globally. At the end of 2020, there were 7.8 million women alive who were diagnosed with breast cancer in the past five years, making it the world's most prevalent cancer [1]. In the majority of cases, breast cancer is a treatable disease and survival rates are still improving thanks to enhancements in screening and treatment [2]. The majority of patients with breast cancer undergo surgery at some point during their treatment. Breast surgery-specific complications can compromise quality of life, increase costs, and delay administration of adjuvant treatment. The most common complication after breast surgery is seroma. Hematoma, surgical site infection (SSI), and chronic neuropathic postoperative pain are other well-known complications [3].

The postoperative SSI rate after breast cancer surgery varies between 0 and 19% [4]. In a review by O'Connor et al., based on 99 studies and almost 500 000 patients, the mean incidence for postoperative SSI was 13.1%. The most common causative bacteria were S. aureus, E. coli, and P. aeruginosa [5]. Known factors that influence the rate of SSI are high age, obesity, diabetes, current or recent smoking, previous chest irradiation, and recent chemotherapy. Hypertension, ASA score 3 or 4, a history of previous breast surgery, hematoma, seroma, more intraoperative bleeding, transfusion, postoperative drain, longer drainage time, second drainage tube placed, insertion of a breast implant or tissue expander, suboptimal prophylactic antibiotic dosing, and lengthy or bilateral procedures have also been reported to increase the risk [3, 6, 7]. There are many reasons to try to reduce the risk for SSI after breast cancer surgery. For example, SSI can delay the start of adjuvant treatment, cause morbidity, increase health care costs, and lead to reconstruction failure. A study from the USA suggests an incremental cost over $4000 per patient in the event of an SSI [8]. Furthermore, some data suggest that SSI may increase the risk for breast cancer recurrence [5, 9–12], but data is not yet conclusive. Understanding risk factors for SSI after breast cancer surgery is essential in order to develop infection-prevention strategies and improve surgical and maybe even oncological outcomes.

The majority of previous studies on SSI do not classify the results according to breast cancer surgery type, but rather pool all surgical procedures together. The type of surgical procedure is an important aspect when considering SSI, as there are multiple operative factors such as surgical technique, surgery time, damage to lymphatic drainage, and neoadjuvant chemotherapy that may influence the risk of an SSI [5]. The aim of the current study was to assess risk factors for SSI, including type of breast/axillary surgery performed. A second aim was to examine the risk factors for other postoperative wound complications such as wound dehiscence, skin necrosis, hematoma, and flap failure.

## 2. Materials and Methods

### 2.1. Data Collection

According to clinical routine, and as part of an internal quality-control project, wound complications were prospectively registered into forms with specified prechosen variables for breast cancer patients undergoing surgery at Uppsala University Hospital May 2017-May 2019. On the day of surgery, the surgeon filled out the first form, including the following variables: patient characteristics, reoperation, type of surgery in the breast/axilla, antibiotic prophylaxis, main operator/assistant, surgery time and breast specimen weight (only second year). The second form was filled out at the postoperative clinical appointment at approximately three weeks, by the breast nurse or the surgeon. The second form included information on wound culture results (if taken), treatment of SSI, wound dehiscence, skin necrosis, hematoma requiring surgery, reoperation, seroma, and aspiration of seroma. The third form was filled out by the oncologist at the patients' first postoperative visit to the oncology department, approximately four weeks after surgery. The variables entered by the oncologist were wound culture results (if taken), treatment of SSI, wound dehiscence, skin necrosis, and hematoma requiring surgery, and also if antibiotics were prescribed prophylactically for those planned for adjuvant treatment. If there were missing data of the preset variables on the forms, the patient's electronic records were scrutinized to complete the data. In the patient charts/forms, unreasonably, few had a noted diagnosis of seroma, and this data was thus deemed unreliable. The postoperative follow-up was a minimum of 30 days or until reoperation, which ever came first. The article was written in accordance with the STROBE guidelines [13].

### 2.2. Patients

In this prospectively registered cohort study, patients who underwent breast-conserving surgery (BCS) or mastectomy for breast cancer between May 2017 and May 2019 were included. Patients with bilateral surgery were registered as two separate operations, and those subjected to reoperation were registered twice, with the exception of reoperation within 24 hours which was evaluated as one single procedure.

### 2.3. Outcome and Predictors

The outcome was dichomotized as SSI/wound complication (SSI, wound dehiscence, skin necrosis, hematoma requiring surgery, and flap failure) or no SSI/wound complication within 30 days from surgery. An SSI was defined as at least one of the following: (1) purulent discharge, (2) positive wound culture, or (3) treatment with antibiotics, drainage, or incision in conjunction with at least one of (A) increasing erythema, (B) local heat and swelling, or (C) increasing pain. White blood cell count and C-reactive protein were not routinely measured to diagnose SSI. Wound culture was routinely taken if an SSI was suspected.

The primary outcome was to examine risk factors for SSI and the secondary outcome risk factors for other wound complications. Factors analyzed were age at the time of surgery, body mass index (BMI), smoking status, diabetes, baseline surgery, type of breast and/or axillary surgery, antibiotic prophylaxis, main surgeon, assisting surgeon, reoperation within 24 hours, operation time, and breast specimen weight. According to the clinical routine at that time, all patients who underwent mastectomy received a drainage until the next day whereas patients subjected to breast conserving surgery did not. For this reason we did not include drainage as a risk factor, as it was already included in the surgical approach. Neither did we include neoadjuvant chemotherapy since it was not a risk factor for SSI in a previous cohort study at our department [14]. Antibiotic prophylaxis was not given routinely but was given to those considered to have a higher risk to suffer an SSI, i.e., patients with neoadjuvant chemotherapy, reoperation within 30 days from baseline surgery, with reconstruction, axillary clearance, operation duration of more than 90 minutes, or patients with specific risk factors. The baseline surgery was either the primary breast surgery for patients or reoperation due to sentinel lymph node biopsy (SLNB) after primary surgery, subsequent axillary clearance due to SLNB macrometastasis, reoperative breast surgery due to nonradical initial surgery, breast surgery after neoadjuvant treatment (SLNB prior to), or subsequent prophylactic mastectomy.

### 2.4. Statistical Analysis

Descriptive data are presented as numbers with percentages and mean (SD). The association between predictors and outcome was analyzed using simple logistic regression. Multiple logistic regression was performed to adjust for confounding predictors. Factors that proved significant on simple analysis were included in multiple analysis. Results are presented as odds ratios (OR) with 95 percent confidence intervals (CI). All analyses were performed using SPSS® version 25 (IBM, Armonk, New York, USA). All statistical tests were two-tailed and a *P* value < 0.05 was considered statistically significant.

### 2.5. Ethical Considerations

The study was approved by the Regional Ethical Committee at Uppsala University (DNR.2018/312).

## 3. Results

The study cohort consisted of 592 patients who underwent 707 procedures. Of those, 26 were bilateral procedures. Nineteen patients were followed less than 30 days due to reoperation within 30 days (median 27 days). Three of these had an SSI before reoperation, and no one had another wound complication before reoperation.

Patient characteristics are shown in Table 1 and treatment variables in Table 2. Mean age was 62 ± 13 years, ranging from 24 to 96 years. Mean BMI was 26.2 ± 4.7, ranging from 16.0 to 47.0. Mean surgery time was 82 ± 54 minutes, ranging from eight to 564 minutes, and mean breast specimen weight was 193 ± 300 gram, ranging from four to 1566 gram.

Complications are shown in Table 3. Of the 707 surgeries there were 66 (9.3%) SSI and 95 (13.4%) wound complications. Mean time to SSI was 17.4 days, ranging from two to 36 days. Wound culture was performed on 39 (59.1%) of the patients with SSI. Thus, twenty-five of the patients with a clinical SSI (37.9%) had a proven positive wound culture. Fifty-five patients with SSI were treated with antibiotics, six with antibiotics and drainage, and two with antibiotics and surgery. Three patients were treated conservatively with dressing and clinical control. Two of these three had positive wound culture. Sixteen patients had a reoperation within 24 hours, fifteen due to hematoma, and one due to free flap failure.

Infection and wound complication rates according to type of breast and axillary surgery are shown in Table 4. After BCS, oncoplastic BCS and doughnut mastopexi with SLNB 9.2%, 19%, and 2.3%, respectively, had an SSI. After mastectomy alone, 25.0% had an SSI. Patients undergoing axillary clearance suffered from SSI in 9.9%. After mastectomy without reconstruction and with immediate reconstruction 19.5% and 32.4%, respectively, presented with a wound complication.

On unadjusted analysis, BMI > 25, oncoplastic BCS, reoperation within 24 hour, and prolonged operative time (90-120 minutes) were significantly risk factors for SSI. BMI > 25 was the only predictor remaining a significant risk factor for SSI on multiple regression analysis (Table 5).

Risk factors for wound complications were almost the same as for SSI (Table 6, supplemental files), except type of breast surgery. On adjusted analysis, mastectomy without reconstruction (OR 2.27, *P* = 0.006) and mastectomy with immediate reconstruction (OR 4.42, *P* = 0.008) were significant risk factors in addition to BMI 25-30 (OR 1.75, *P* = 0.036) and BMI > 30 (OR 1.93, *P* = 0.032) for any wound complication.

## 4. Discussion

In this prospective single-center study, the SSI and wound complication frequency were 9.3% and 13.4%, respectively. This is consistent with previous studies [4, 5]. In the unadjusted analysis, risk factors for SSI were BMI > 25, oncoplastic BCS, reoperation within 24 hours, and surgery time 90-120 minutes. Only BMI > 25 remained a risk factor on adjusted analysis although there was a trend towards more SSI after oncoplastic BCS (HR 2.02, *P* = 0.084). Obesity (BMI > 30) is a known risk factor for SSI [3, 6, 15]; however, the current study shows that simple overweight (BMI 25-30) also was associated with a doubled risk. A potential reason for this could be that overweight/obesity would reduce the actual dose of prophylactic antibiotics due to the increased mass, since antibiotic penetration into fat is relatively poor [16]. For example, Olsen et al. describe that receiving a suboptimal dose of prophylactic antibiotic is associated with 5.1-fold increased odds of breast SSI 7].

Other risk factors for wound complications included mastectomy, especially in combination with immediate reconstruction. In reconstructive and oncoplastic surgery, extensive tissue manipulation is common, which can damage the blood supply of the flaps and cause necrosis and flap dehiscence [3]. Good surgical technique, based on adequate training and knowledge of the blood supply of flaps, is necessary in order to avoid these complications. Reoperation for surgical bleeding is more common after mastectomy than after BCS [17]. Seroma is much more common after mastectomy than after BCS [18]. Hematoma and seroma after surgery are known risk factors for SSI [6] and is likely even for other wound complications; this can contribute to the increased risk for wound complications after mastectomy. So adequate hemostasis, handling of tissue gently and closing the incision without tension are important to avoid complications.

In the current study, there was a trend towards an association between breast specimen weight > 100 g and the risk for wound complication (OR 7.07, *P* = 0.062), which is in line to findings by Ito et al. that showed that breast specimen weight was an important risk factor for skin flap necrosis in immediate reconstruction [19]. The failure to reach significance regarding this association may be explained by a small sample size for this outcome, since this study only included 37 patients with immediate reconstruction and not all specimens were weighed.

Many of previously described risk factors for SSI were not significant risk factors in the current study. Age, diabetes, and smoking are known factors that can influence the rate of SSI [3]. In this study, age and smoking were not risk factors for SSI. There was a trend for patients with diabetes to have higher risk for SSI. In a study by Valente et al. [15], these three predictors neither were risk factors for wound complications following mastectomy with immediate reconstruction. In a meta-analysis, smoking habit was not a significant risk factor for SSI but diabetes was [6]. Even though many studies show that smoking is a risk factor for SSI [3, 7], there are conflicting evidence. In this study, only 73 patients were current smokers which can have affected that no correlation was found between smoking and SSI.

Axillary clearance has previously been shown to be associated with SSI [11, 14], but this association was not seen in the current cohort. In a previous study by our group on a cohort from year 2009 and 2010, the incidence was three times higher than for breast surgery without axillary surgery [14]. The difference between the two cohorts may be explained by the fact that the clinical routine for antibiotic prophylaxis has changed and that such prophylaxis was administered to all patients undergoing axillary clearance in the later time period but only in few selected cases in the 2009-2010 period. The absolute percentage of patients with SSI of those undergoing axillary clearance in the 2009-2010 cohort in comparison to this cohort was (39/176) 22.2% vs. (11/111) 9.9%.

The SSI frequency was low (<10%) for BCS, in particular doughnut mastopexy, even though they did not receive antibiotic prophylaxis. The SSI frequency was considerably higher for oncoplastic BCS and mastectomy alone had the highest SSI rate. The high rate of SSI in the mastectomy only group was probably heavily influenced by confounding, as only the most fragile patients, not suitable for reconstruction and/or axillary surgery would have been selected for mastectomy alone according to the prevailing clinical routine. Another fact contributing to these results may be that patients undergoing reconstruction and/or axillary clearance received antibiotic prophylaxis, which was not the case if mastectomy alone was performed.

Xue et al. showed in a meta-analysis that the benefit from antibiotic prophylaxis was not significant [6]. However, in a cohort study, the rate of SSIs decrease significantly based on the administration of antibiotic prophylaxis to high-risk patients only [20]. After adjusting for confounding factors, antibiotic prophylaxis reduced the risk for SSI in breast cancer surgery by 81% in the high-risk group. They suggest that antibiotic prophylaxis should not be given on routine but to patients if risk factors are accompanied. Based on the current study, patients with BMI > 25 (overweight) and patients undergoing oncoplastic breast surgery probably have most benefit from antibiotic prophylaxis in addition to already known risk factors as obesity, neoadjuvant chemotherapy, reoperation, reconstruction, axillary clearance, surgery lasting > 90 minutes, and patients with specific risk factors.

Since overweight/obesity was the only modifiable significant predictor for developing SSI/wound complication after breast cancer surgery, it is important to take that into consideration when counseling women with breast cancer. Patients need to be aware that BMI > 25 can increase the risk for postoperative complications, delay their treatment time [15], and maybe even increase the risk for breast cancer recurrence [5, 9–12]. The risk for complications increases with the degree of obesity. Delayed reconstruction after intentional weight loss is an option that could be offered to patients with overweight/obesity. Mastectomy is not a good option if BCS is possible since postoperative complications are higher and some studies even suggest that the oncological outcome is worse [21–26].

Breast cancer surgery is thought to be clean surgery but has higher SSI frequency than suggested for clean surgery. One theory is that the cancer itself may play a role in the development of SSI. In a study by Olsen et al., patients undergoing breast cancer surgery had increased risk for SSI compared with those undergoing breast reduction surgery due to macromastia despite they are very similar surgical procedures in term of duration and length of incisions [7].

Currently, there is no worldwide “golden standard” for the diagnosis of SSI. In a review by O'Connor et al., there was notable variation in criteria used to define SSI and it was not defined at all in 45% of the studies [5]. The strength of this study is that is it prospective, and the SSI definition was clear. The recommendation was to take a wound culture, notwithstanding it was sometimes difficult to establish a clear SSI diagnosis. In case of fever and erythema of the breast but no discharge or seroma, a wound culture is also not possible. One limitation is that not all patients had a wound culture. Even though the recommendation was to take wound culture, it was done in 59.1% (*n* = 39) of patients with clinical SSI diagnosis and 64.1% (*n* = 25) of those had a positive culture. Compared to the review by O'Connor et al., this is in all cases higher since only 10% of the studies used a culture-positive result to establish an SSI [5]. Another limitation is that tests for white blood cell count or C-reactive protein were not taken on routine. These tests could possibly be helpful in the diagnosis of a SSI; however, due to logistical limitations of the outpatient clinic, it was rarely done. However, factoring the arguments above, the frequency of SSI is probably more reliable in this prospective study than in retrospective studies [5]. A further limitation is that not all previous suggested risk factors were registered in the form such as drainage and neoadjuvant chemotherapy. The reason we chose not to include neoadjuvant chemotherapy was because it was not a risk factor for SSI in a previous cohort studies at our department 2009-2010 HR (CI) 1.53 (0.43, 5.47) [14]. Seroma was unfortunately not assessed in any validated or quantitative way (such as ultrasound) during the study period. In the patient charts/forms, unreasonably, few had a noted diagnosis of seroma, and this data was thus deemed unreliable. Of that reason, seroma formation was not possible to analyze and represents another limitation. Omission of this data may theoretically have influenced the results of this study.

Eighty-three percent of the patients with SSI were treated only with antibiotics, and three patients were conservatively treated. Although SSI often is a minor complication easily dealt with, there are many reasons to reduce this complication. SSI can delay start of adjuvant treatment, cause morbidity, increase costs, and lead to failed reconstructions. Furthermore, some data suggest that SSI may increase the risk for breast cancer recurrence [5, 9–12].

## 5. Conclusions

The present study confirms that BMI > 30 is a strong risk factor for SSI and other wound complications, but even that BMI 25-30 is associated with a doubled risk of SSI. Mastectomy with or without immediate reconstruction were both risk factors for wound complications, and there was a trend for oncoplastic BCS as a risk factor for SSI. Knowledge of these risk factors is of most importance to take actions for patients to reduce the risk for SSI and other wound complications with the goal to improve outcome, decrease morbidity and cost in breast cancer patients. Antibiotic prophylaxis should be given on individual basis and must be specially tailored to the needs of overweight/obese patients to provide adequate tissue levels. In addition, carefully patient selection concerning immediate reconstruction and oncoplastic BCS is of utmost importance.

## Figures and Tables

**Table 1:** Patient characteristics.

Patient variables	Total cohort (*n* = 707)
Age at surgery (years)	
**≤**46	79 (11.2)
46-60	228 (32.2)
61-74	299 (42.3)
>74	101 (14.3)
BMI (kg/m^2^)	
<18.5	11 (1.6)
18.5-24.9	309 (43.7)
25-30	247 (34.9)
>30	138 (19.5)
Missing data	2 (0.3)
Tobacco users	
No	627 (88.7)
Yes	73 (10.3)
Missing data	7 (1.0)
Diabetic	
No	668 (94.5)
Yes	39 (5.5)

Values are number (percent).

**Table 2:** Treatment characteristics of study cohort.

Treatment variables	Total cohort (*n* = 707)
Baseline surgery is primary surgery	
No	98 (13.9)^#^
Yes	609 (86.1)
Breast surgery	
BCS	292 (41.3)
Oncoplastic BCS	99 (14)
Doughnut mastopexy	66 (9.3)
Mastectomy no reconstruction	154 (21.8)
Immediate reconstruction	37 (5.2)^##^
Only axillary surgery	59 (8.3)
Axillary surgery	
Sentinel lymph node biopsy	414 (58.6)
Axillary clearance	111 (15.7)
Axillary sampling	18 (2.5)
Only breast surgery	164 (23.2)
Antibiotic prophylaxis	
No	335 (47.4)
Yes	327 (46.3)
Missing data	45 (6.4)
Main operator	
Breast surgeon	604 (85.4)
Resident/surgeon (other subspeciality)+BS assist	83 (11.7)
Surgeon (other subspeciality)	20 (2.8)
Assistant	
Only scrub nurse	266 (37.6)
One assistant	404 (57.1)
Two assistants	37 (5.2)
Reoperation within 24 hours	
No	691 (97.7)
Yes	16 (2.3)
Surgery time (minutes)	
<60	242 (34.2)
60-89	229 (32.4)
90-120	153 (21.6)
>120	83 (11.7)
Breast specimen weight (gram)	
<16	34 (4.8)
16-50	108 (15.3)
51-100	55 (7.8)
>100	102 (14.4)
Missing data	408 (57.7)

Values are number (percent). BCS: breast-conserving surgery; SLNB: sentinel lymph node biopsy; BS: breast surgeon; Assist: assistant. ^#^SLNB after primary surgery (*n* = 14), subsequent axillary clearance because of SLNB macrometastasis (*n* = 9), reoperation due non radicalinitial surgery (*n* = 41), combination of the two before (*n* = 3), breast surgery after neoadjuvant treatment (SLNB prior to) (*n* = 29), subsequent prophylactic mastectomy (*n* = 2). ^##^Expander (*n* = 16), implant (*n* = 4), implant+acellular dermal matrix (*n* = 2), deep inferior epigastric perforator flap (*n* = 11), goldilocks procedure (*n* = 4).

**Table 3:** Complication rates.

Complication	Total cohort, (*n* = 707)
SSI	66 (9.3)
Wound dehiscence	25 (3.5)
Skin necrosis	16 (2.3)
Hematoma (requiring surgery)	15 (2.1)
Flap failure	1 (0.1)
Wound complication^#^	95 (13.4)

Values are number (percent). SSI: surgical site infection. ^#^SSI, wound dehiscence, skin necrosis, hematoma requiring surgery, flap failure.

**Table 4:** Infection and wound complication rates.

Type of breast/axillary surgery	SSI	Wound complication^#^
BCS (*n* = 292)	23 (7.9)	28 (9.6)
No axillary surgery (*n* = 65)	3 (4.6)	3 (4.6)
SLNB (*n* = 196)	18 (9.2)	23 (11.7)
Axillary clearance (*n* = 28)	2 (7.1)	2 (7.1)
Axillary sampling (*n* = 3)	0 (0.0)	0 (0.0)

Oncoplastic BCS (*n* = 99)	15 (15.2)	16 (16.2)
No axillary surgery (*n* = 25)	2 (8.0)	3 (12.0)
SLNB (*n* = 58)	11 (19.0)	11 (19.0)
Axillary clearance (*n* = 15)	2 (13.3)	2 (13.3)
Axillary sampling (*n* = 1)	0 (0.0)	0 (0.0)

Doughnut mastopexi (*n* = 66)	1 (1.5)	6 (9.1)
No axillary surgery (*n* = 14)	0 (0.0)	2 (14.3)
SLNB (*n* = 43)	1 (2.3)	4 (9.3)
Axillary clearance (*n* = 8)	0 (0.0)	0 (0.0)
Axillary sampling (*n* = 1)	0 (0.0)	0 (0.0)

Mastectomy (no reconstr) (*n* = 154)	20 (13.0)	30 (19.5)
No axillary surgery (*n* = 36)	9 (25.0)	9 (25.0)
SLNB (*n* = 64) Axillary clearance (*n* = 42)	5 (7.8) 5 (11.9)	12 (18.8) 8 (19.0)
Axillary sampling (*n* = 12)	1 (8.3)	1 (8.3)

Immediate reconstruction (*n* = 37)	4 (10.8)	12 (32.4)

SLNB (*n* = 414) (regardless of breast surgery)	39 (9.4)	56 (13.5)

Axillary clearance (*n* = 111) (regardless of breast surgery)	11 (9.9)	14 (12.6)

Reoperation (within 24 hours) (*n* = 16)	4 (25)	

Values are number (percent). SSI: surgical site infection; BCS: breast-conserving surgery; SLNB: sentinel lymph node biopsy. ^#^SSI, wound dehiscence, skin necrosis, hematoma requiring surgery, flap failure.

**Table 5:** Unadjusted and adjusted logistic regression analyses of factors associated with SSI.

Factors	No. of patients	Unadjusted results		Adjusted results (*n* = 705)	
		Odds ratio (CI)	*P*	Odds ratio (CI)	*P*
Age at surgery (years) (*n* = 707)					
<46	79	1.00 (reference)			
46-60	228	1.91 (0.71, 5.14)	0.204		
61-74	299	1.41 (0.52, 3.80)	0.497		
>74	101	1.45 (0.47, 4.51)	0.523		
BMI (kg/m^2^) (*n* = 705)					
18.5-24.9	309	1.00 (reference)		1.00 (reference)	
<18.5	11	0.00 (0.00, 0.00)	0.999	0.00 (0.00, 0.00)	0.999
25-30	247	1.90 (1.02, 3.56)	0.044	1.98 (1.05, 3.75)	0.036
>30	138	2.90 (1.49, 5.64)	0.002	2.85 (1.43, 5.67)	0.003
Tobacco user (*n* = 700)					
No	627	1.00 (reference)			
Yes	73	1.04 (0.46, 2.37)	0.925		
Diabetic (*n* = 707)					
No	668	1.00 (reference)			
Yes	39	2.26 (0.96, 5.34)	0.064		
Baseline surgery prime surg (*n* = 707)					
Yes	609	1.00 (reference)			
No	98	0.85 (0.57, 0.26)	0.415		
Breast surgery (*n* = 707)					
BCS	292	1.00 (reference)		1.00 (reference)	
Oncoplastic BCS	99	2.09 (1.04, 4.19)	0.038	2.02 (0.91, 4.51)	0.084
Doughnut mastopexy	66	0.18 (0.02, 1.36)	0.096	0.18 (0.02, 1.36)	0.095
Mastectomy no reconstruction	154	1.75 (0.93, 3.29)	0.085	1.59 (0.81, 3.12)	0.178
Immediate reconstruction	37	1.42 (0.46, 4.35)	0.542	1.62 (0.37, 7.01)	0.521
Only axillary surgery	59	0.63 (0.18, 2.16)	0.459	0.79 (0.22, 2.85)	0.720
Axillary surgery (*n* = 707)					
Sentinel lymph node biopsy	414	1.00 (reference)			
Axillary clearance	111	1.06 (0.52, 2.14)	0.876		
Axillary sampling	18	0.57 (0.07, 4.37)	0.585		
Only breast surgery	164	0.97 (0.52, 1.81)	0.919		
Antibiotic prophylaxis (*n* = 662)					
No	335	1.00 (reference)			
Yes	327	1.31 (0.78, 2.22)	0.305		
Main operator (*n* = 707)					
Breast surgeon	604	1.00 (reference)			
Resid/Surg (other subspec)+BSassist	83	1.07 (0.49, 2.32)	0.875		
Surgeon (other subspec)	20	1.76 (0.50, 6.20)	0.378		
Assistant (*n* = 707)					
Only scrub nurse	266	1.00 (reference)			
One assistant	404	1.15 (0.67, 2.00)	0.615		
Two assistants	37	2.15 (0.87, 5.70)	0.125		
Reoperation (within 24 hours) (*n* = 707)					
No	691	1.00 (reference)		1.00 (reference)	
Yes	16	3.38 (1.06, 10.80)	0.040	2.67 (0.79, 9.10)	0.115
Surgery time (minutes) (*n* = 707)					
<60	242	1.00 (reference)		1.00 (reference)	
60-89	229	1.73 (0.86, 3.47)	0.122	1.45 (0.69, 3.03)	0.322
90-120	153	2.59 (1.28, 5.27)	0.009	1.71 (0.75, 3.88)	0.202
>120	83	1.98 (0.82, 4.76)	0.127	1.18 (0.37, 3.74)	0.782
Breast specimen weight (g) (*n* = 299)					
<16	34	1.00 (reference)			
16-50	108	2.64 (0.32, 21.90)	0.368		
51-100	55	6.46 (0.78, 53.46)	0.084		
>100	102	3.99 (0.50, 32.11)	0.194		

SSI: surgical site infection; BCS: breast-conserving surgery; prime surg: primary surgery; Resid/Surg: resident/surgeon; BS: breast surgeon; subspec: subspeciality; Assist: assistant; g: gram.

## Data Availability

The data that support the findings of this study are not publicly available due to the ethical restrictions. However, the data may be made available for review upon request to the authors.

## Abbreviations

SSI: Surgical site infection
BCS: Breast conserving surgery
BMI: Body mass index
SLNB: Sentinel lymph node biopsy
OR: Odds ratios
CI: Confidence intervals
BS: Breast surgeon
Assist: Assistant
Prime surg: Primary surgery
Resid/Surg: Resident/surgeon
Subspec: Subspeciality
g: Gram.

## Additional Points

*Synopsis*. In this prospectively registered cohort study, we wanted to examine risk factors for surgical site infection (SSI) and other postoperative wound complications. The present study confirms that BMI > 30 is a strong risk factor for SSI and other wound complications, but even that BMI 25-30 is associated with a doubled risk of SSI. Mastectomy with or without immediate reconstruction were both risk factors for wound complications, and there was a trend for oncoplastic breast conserving surgery as a risk factor for SSI.

## Supplementary Materials

Supplementary Materials Table 6: unadjusted and adjusted logistic regression analyses of factors associated with wound complication.
