# Correction to “Prospective Evaluation of Complications and Associated Risk Factors in Breast Cancer Surgery”

**DOI:** 10.1155/jo/9805269

**Published:** 2026-06-20

L. Adwall, H. Hultin, M. Mani, and O. Norlén, “Prospective Evaluation of Complications and Associated Risk Factors in Breast Cancer Surgery,” *Journal of Oncology*, 2022, https://doi.org/10.1155/2022/6601066.

In the article, there is an error in the limitations paragraph of the Discussion section:

“The reason we chose not to include neoadjuvant chemotherapy was because it was not a risk factor for SSI in a previous cohort studies at our department 2009‐2010 HR (CI) 1.53 (0.43, 5.47) [14].”

should read:

“The reason we chose not to include neoadjuvant chemotherapy was because it was not a risk factor for SSI in a previous study at our department 2014‐2015 OR (CI) 0.96 (0.08,11.00) [unpublished data].”

We apologize for this error.

