# Supplementary material for: Prospective Evaluation of Complications and Associated Risk Factors in Breast Cancer Surgery
Source: J Oncol. 2022 Sep 17;2022:6601066. doi: 10.1155/2022/6601066 (PMC9783023; doi:10.1155/2022/6601066)
Supplement: Supplementary Materials — Table 6: unadjusted and adjusted logistic regression analyses of factors associated with wound complication. [file 6601066.f1.docx]

**Table 6** Unadjusted and adjusted logistic regression analysis of factors associated with wound complication

|  |  | Unadjusted results | | Adjusted results (n=705) | |
| --- | --- | --- | --- | --- | --- |
| Factors | **No of patients** | **Odds ratio (CI)** | **P** | **Odds ratio (CI)** | **P** |
| Age at surgery (years) (n=707)  <46  46-60  61-74  >74 | 79  228  299  101 | 1.00 (reference)  1.47 (0.70, 3.10)  0.92 (0.43, 1.94)  0.76 (0.30, 1.92) | 0.313  0.816  0.560 |  |  |
| BMI (kg/m^2^) (n=705)  18.5-24.9  <18.5  25-30  >30 | 309  11  247  138 | 1.00 (reference)  0.00 (0.00, 0.00)  1.63 (0.98, 2.71)  1.89 (1.06, 3.36) | 0.999  0.059  **0.031** | 1.00 (reference)  0.00 (0.00, 0.00)  1.75 (1.04, 2.95)  1.93 (1.06, 3.52) | 0.999  **0.036**  **0.032** |
| Tobacco user (n=700)  No  Yes | 627  73 | 1.00 (reference)  1.03 (0.51, 2.08) | 0.943 |  |  |
| Diabetic (n=707)  No  Yes | 668  39 | 1.00 (reference)  1.44 (0.62, 3.37) | 0.398 |  |  |
| Baseline surgery prime surg (n=707)  Yes  No | 609  98 | 1.00 (reference)  1.20 (0.66, 2.17) | 0.559 |  |  |
| Breast surgery (n=707)  BCS  Oncoplastic BCS  Doughnut mastopexy  Mastectomy no reconstruction  Immediate reconstruction  Only axillary surgery | 292  99  66  154  37  59 | 1.00 (reference)  1.82 (0.94, 3.52)  0.95 (0.37, 2.38)  2.28 (1.31, 3.98)  4.53 (2.05, 9.98)  0.51 (0.15, 1.72) | 0.077  0.901  **0.004**  **0.000**  0.275 | 1.00 (reference)  1.71 (0.81, 3.59)  0.75 (0.27, 2.09)  2.27 (1.27, 4.06)  4.42 (1.46, 13.36)  0.60 (0.17, 2.09) | 0.158  0.584  **0.006**  **0.008**  0.418 |
| Axillary surgery (n=707)  Sentinel lymph node biopsy  Axillary clearance  Axillary sampling  Only breast surgery | 414  111  18  164 | 1.00 (reference)  0.92 (0.49, 1.73)  0.38 (0.05, 2.88)  1.10 (0.64, 1.84) | 0.801  0.347  0.728 |  |  |
| Antibiotic prophylaxis (n=662)  No  Yes | 335  327 | 1.00 (reference)  1.59 (1.02, 2.50) | **0.042** |  |  |
| Main operator (n=707)  Breast Surgeon  Resid/Surg (other subspec) + BSassist  Surgeon (other subspec) | 604  83  20 | 1.00 (reference)  0.90 (0.45, 1.81)  2.18 (0.77, 6.17) | 0.762  0.141 |  |  |
| Assistant (n=707)  Scrub nurse  One assistant  Two assistants | 266  404  37 | 1.00 (reference)  1.25 (0.78, 1.99)  1.77 (0.72, 4.37) | 0.358  0.216 |  |  |
| Surgery time (minutes) (n=707)  <60  60-89  90-120  >120 | 242  229  153  83 | 1.00 (reference)  1.61 (0.88, 2.94)  2.38 (1.28, 4.41)  3.30 (1.66, 6.55) | 0.121  **0.006**  **0.001** | 1.00 (reference)  1.28 (0.68, 2.41)  1.46 (0.72, 2.96)  1.37 (0.53, 3.54) | 0.440  0.290  0.514 |
| Breast specimen weigh (g) (n=299)  <16  16-50  51-100  >100 | 34  108  55  102 | 1.00 (reference)  3.37 (0.42, 27.31)  7.33 (0.89, 60.13)  7.07 (0.91, 55.13) | 0.256  0.063  0.062 |  |  |

SSI; Surgical Site Infection, BCS; Breast-Conserving Surgery, prime surg; primary surgery, Resid/Surg; Resident/Surgeon, BS; Breast surgeon; subspec; subspeciality, Assist; Assistant, g;gram
